# Using Path Analysis and Linear Regression to Test for Gender and Participation: Effects in a Culturally Tailored Diabetes Intervention for Latino Adults

**DOI:** 10.3390/ijerph191911982

**Published:** 2022-09-22

**Authors:** Jaclynn Hawkins, Edith C. Kieffer, Brandy Sinco, Gretchen Piatt, Lenette Jones, Jamie Mitchell, Nicolaus Espitia, Alana LeBron, Katherine A. Kloss, Katie Kurnick, Gloria Palmsiano, Michael S. Spencer

**Affiliations:** 1 School of Social Work, University of Michigan, 1080 South University Avenue, Ann Arbor, MI 48109, USA; 2 Center for Healthcare Outcomes & Policy, University of Michigan, 2800 Plymouth Road, North Campus Research Complex, Bldg. 16, Ann Arbor, MI 48109, USA; 3 Department of Learning Health Sciences, School of Medicine, University of Michigan, 1111 E. Catherine Street, Ann Arbor, MI 48109, USA; 4 School of Nursing, University of Michigan, 400 North Ingalls Building, Ann Arbor, MI 48109, USA; 5 Department of Sociology, Anthropology, Social Work and Criminal Justice Oakland University, 614 Pioneer Dr, Rochester, MI 48309, USA; 6 School of Public Health, University of California, Irvine, 3151 Social Science Plaza, SST 369 (Chicano/Latino Studies) OR 653 E Peltason Drive, AIRB 2026 (Public Health), Irvine, CA 92697, USA; 7 Community Health and Social Services Center, 5635 West Fort Street, Detroit, MI 48209, USA; 8 School of Social Work, University of Washington, 4101 15th Avenue NE, Seattle, WA 98105, USA

**Keywords:** diabetes, gender, self-management, Latino, path analysis, intervention

## Abstract

While the incidence and prevalence of type 2 diabetes is higher among Latino/as, Latino men are disproportionately affected and have poorer outcomes. We aimed to determine whether gender impacted any outcomes in a culturally tailored type 2 diabetes (T2D) intervention and to evaluate the effects of gender and intervention participation intensity on outcomes at 6-month follow-up. Nested path and regression models were compared with the likelihood ratio test and information criteria in a sample of Latino/a adults with T2D (*n* = 222) participating in a T2D community health worker (CHW)-led intervention. Path analysis showed that the effect of the intervention did not vary by gender. The intervention was associated with significant improvements in knowledge of T2D management 0.24 (0.10); *p* = 0.014, diabetes distress, −0.26 (0.12); *p* = 0.023, and self-efficacy, 0.61 (0.21); *p* = 0.005. At 6-month follow-up, improved self-management was associated with greater self-efficacy and Hemoglobin A1c (HbA1c) was lower by −0.18 (0.08); *p* = 0.021 for each unit of self-management behavior. Linear regressions showed that class attendance and home visits contributed to positive intervention results, while gender was non-significant. Pathways of change in a CHW-led culturally tailored T2D intervention can have a significant effect on participant behaviors and health status outcomes, regardless of gender.

## 1. Introduction

An estimated 37.3 million adults aged 18 years or older have diabetes, representing 11.3% of all U.S. adults [1]. Of these 37.3 million, 35.4 million have type 2 diabetes (T2D). Overall, the prevalence of T2D is higher among people of Hispanic ethnicity (11.8%) when compared to non-Hispanic whites (7.4%) and Asians (9.5%) [1]. Further, 40% of US adults are expected to develop T2D during their lifetime [1,2]. Among all Hispanics, males (13.7%) have a higher prevalence rate of diagnosed T2D than females (11.6%) [1]. This lifetime prevalence rises to 50% for Latino men and women [1]. In addition to higher prevalence rates, Latino adults are disproportionately more likely to suffer from long-term diabetes complications such as chronic kidney disease, stroke, and cardiovascular disease compared to non-Hispanic whites [1,2].

Community-based participatory research (CBPR) partnerships were developed, implemented, and evaluated as one potential avenue for addressing the T2D epidemic among Latinos in the U.S. [3,4]. Specifically, several CBPR efforts focused on improving the efficacy of community health worker (CHW) interventions to support diabetes self-management behaviors [5,6,7,8,9,10]. CHWs are lay individuals trained in goal setting, problem solving, and providing ongoing social and emotional support for diabetes self-management, particularly in diverse communities [5,8,9,10,11]. CHWs often share cultural identity and community ties with those they support, and this is considered to be a critical component in fostering trust with the diverse clients they support with T2D [5,11,12]. While extant research suggests that Latino men are less engaged in CHW initiatives [13], few studies specifically investigate this lack of participation or highlight efforts to better recruit and retain Latino men in CHW intervention research targeting T2D. The current body of knowledge on diabetes-focused CHW programs also neglects attention to gender disparities in the utilization and efficacy of such interventions. However, much can still be gleaned from studies that have successfully utilized lay helper models to explicitly target Latino men in the promotion of sexual health, such as the HoMBReS program developed by Rhodes et al. [14,15]. The Hombres Manteniendo Bienestar y Relaciones Saludables (Men Maintaining Wellbeing and Healthy Relationships) (HoMBReS) intervention provided evidence that strategies involving lay health advisors can decrease risk behavior. In the case of HoMBRes, utilization of lay health advisors (or Navigantes) in conjunction with community social networks led to an increase in condom use and HIV testing among Hispanic/Latino men [14,15]. Testing and evaluation of evidence-based lay helper models are critical to developing sustainable strategies to reach Latino men.

Attention to gender in the development of CHW interventions is particularly important because Latino men often face different challenges to engaging in managing their diabetes and interacting with health care systems compared to Latina women, potentially increasing their risk for diabetes-related complications [16]. Research confirms that gender norms, such as holding certain beliefs about being a man, can serve as either barriers or facilitators to supporting glycemic control in Latino men [17]. The degree to which gender differences influence change in glycemic control among participants in a CHW diabetes lifestyle intervention for Latino/as remains understudied. Therefore, we sought to address this gap in knowledge by identifying gender differences in the effect of culturally tailored CHW-led diabetes self-management intervention that improved glycemic control among Latino adults with T2D. We further examined the overall effects of intervention components (class attendance, group format, home visits, doctor visits) on intermediate outcomes and glycemic control.

## 2. Materials and Methods

The study analyzes data from the RCT conducted between October 2009 and February 2013 with Latino/a adults by the Racial and Ethnic Approaches to Community Health (REACH) Detroit Partnership, as part of the Centers for Disease Control and Prevention (CDC)-funded initiative, utilizing community-based participatory research (CBPR) principles [6,7,8,9,10]. Latino/a adults with T2D (*n* = 222) from a Federally Qualified Health Center in Detroit were randomized to a CHW-led diabetes education intervention, or an enhanced usual care control group. Survey and lab data were collected at baseline and 6-month follow-up. The hypothesis being tested was that the intervention would impact diabetes distress, diabetes self-efficacy, knowledge of diabetes self-management, which would improve self-management behavior (defined below), and then subsequently improve glycemic control at 6-month follow-up.

The main outcome was Hemoglobin A1c (A1c). Other outcomes included diabetes self-management behavior, diabetes distress, diabetes self-efficacy, knowledge of diabetes self-management. Diabetes self-management behavior was assessed with the Summary of Diabetes Self-Care Activities (SDSCA) scale [18]. Diabetes distress was measured by the Diabetes Distress Scale [19]. The Stanford Self-Efficacy for Diabetes was used to estimate self-efficacy [20]. Last, the Diabetes Knowledge Test was used to assess knowledge of diabetes self-management [21,22]. Intervention participation was assessed by the number of home visits from a community health worker, intervention class attendance, and whether the client attended all of part 1 or part 2 intervention classes in group format.

Demographic characteristics included binary gender (female or male), high school graduate (yes or no), and age in years. Diabetes medication intensification was a binary variable to indicate whether the number of medications or the dosages increased from baseline to 6-month follow-up.

Path analysis was used to test whether gender, treatment group, and interactions between gender and treatment group impacted any outcomes. Linear regression was used on the intervention subset (*n* = 123) to assess the effects of gender, intervention participation, and possible interaction. Nested path and regression models were compared with the likelihood ratio test and information criteria to determine model fit.

The path analysis model was estimated by FIML (Full Information Maximum Likelihood). Path analysis was evaluated with Bentler’s Comparative Fit Index (CFI) for comparative fit, the Steiger–Lind Root Mean Square Error of Approximation (RMSEA) for parsimony, and the (Standardized Root Mean Square Residual) SRMR for prediction. The path analysis model contained 5 equations. Three equations for each of diabetes distress, diabetes self-efficacy, and knowledge of diabetes self-management consisted of the 6-month value as the outcome with inputs of gender, treatment group, gender by treatment group interaction, and baseline value. The next equation had self-management behavior as the outcome with inputs of gender, treatment group, gender by treatment group interaction, baseline self-management behavior, 6-month diabetes distress, diabetes self-efficacy, and knowledge of diabetes self-management. In the final equation, 6-month A1c was the outcome and the covariates were baseline A1c, medication intensification, gender, treatment group, gender by treatment group interaction, age, high school education, and 6-month self-management behavior [23,24,25,26,27,28,29].

The linear regression models were similar to the path models, except that treatment group was excluded because the data contained the intervention subset, and that indicators for participation were added to each model. The equations for each of diabetes distress, diabetes self-efficacy, and knowledge of diabetes self-management consisted of the 6-month value as the outcome with inputs of gender, age, high school education, baseline value, class attendance, all intervention classes in group format, and number of home visits. The self-management behavior equation had inputs of gender, age, high school graduate, baseline self-management behavior, 6-month diabetes distress, diabetes self-efficacy, knowledge of diabetes self-management, along with participation measures. In the A1c equation, the covariates were baseline A1c, medication intensification, gender, age, high school education, and 6-month self-management behavior.

## 3. Results

### 3.1. Sample Demographic and Participation Characteristics by Gender

Table 1 summarizes the demographics and participation of the sample. The ages of the women and men in the study were similar, participants were on average 50 years of age. About 60% of the sample was female and men were more likely to be high school graduates, 40% of men compared to 24% of women. On average, participants attended 7.6 out of 11 intervention classes, with women attending eight classes and men attending seven. More men (62%) attended all healthy lifestyles classes in group format compared to women (43%). Similarly, 73% of men attended all self-management classes in group format compared to 56% of all women. Both men and women received an average of one CHW-accompanied doctor visit.

### 3.2. Path Analysis Results

**Figure 1:**
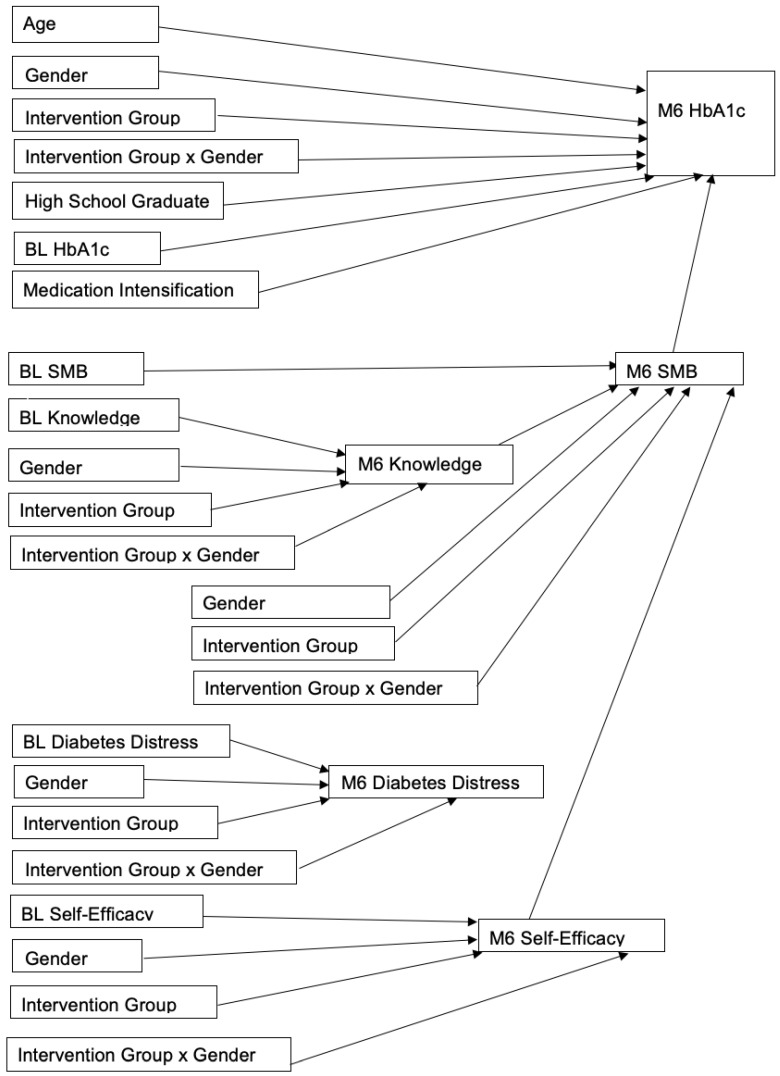
Path Analysis with covariates.

**Figure 2:**
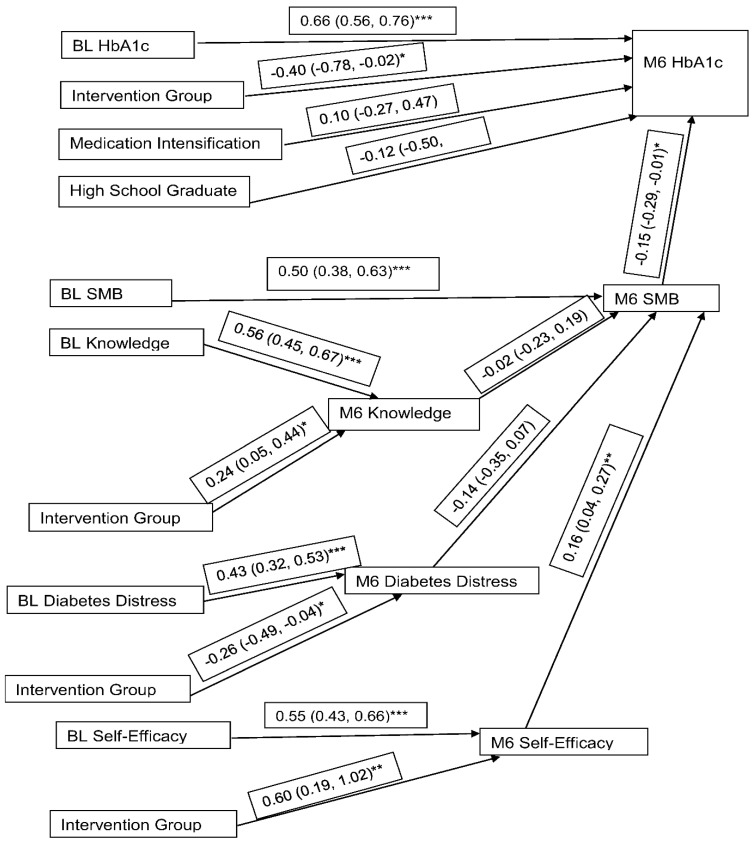
Path Analysis with Model Coefficients and Standard Errors. (* *p* < 0.01, ** *p* < 0.05, *** *p* < 0.001).

The path analysis results are displayed in Figure 1 as coefficient (95% confidence interval) and showed that the effect of the intervention did not vary by gender. Our initial path model is shown in Figure 1, and included demographics age, gender, intervention group, gender by intervention interaction, and education. Because education differed by treatment group at baseline, education was not removed from the model. The likelihood ratio test and information criteria were used to check whether the model fit better with or without age, gender, and the gender by intervention interaction. Because the likelihood ratio test and information criteria indicated that the model fit better without these covariates, they were removed, and we report the results of the more parsimonious model in Figure 2.

All 6-month outcomes were significantly related to their baseline starting values. Based on the path coefficients, being in the intervention group was associated with a 0.60 (0.19, 1.02) increase in diabetes self-efficacy, *p* < 0.01, −0.26 (−0.49, −0.04) reduction in diabetes distress, *p* < 0.05, 0.24 (0.05, 0.44) increase of knowledge of diabetes management, *p* < 0.05, and −0.40 (−0.78, −0.02) drop in A1c, *p* < 0.05.

Higher self-efficacy was significantly associated with better self-management at 6-months. A unit increase in self-efficacy corresponded to an average increase of 0.16 (0.04, 0.27), *p* < 0.01, in self-management behavior. Finally, better self-management behavior lowered 6-month A1c, for each unit increase in self-management behavior, 6-month A1c was reduced by −0.15 (−0.29, −0.01), *p* < 0.05. Finally, based on the model fit indices, the path model had good parsimony, comparative fit, and predictive ability from an RMSEA (90% CI) 0.059 (0.037, 0.079), Bentler Comparative Fit Index of 0.964, and Standardized Root Mean Square Residual (SRMR) of 0.0504. Based on the power calculation, n = 222 corresponds to 80% power. For the path analysis, power was estimated by the method of MacCullum, Browne, and Sugawara (1996). For N = 222, we had 80% power to detect a change in the model chi-square statistic.

### 3.3. Linear Regression Results

As stated, linear regression was used to test for interactions between gender and participation. Gender was not significant in any linear regression models and models fit increased when interactions between gender and participation were removed. Intervention class attendance was a significant predictor of Self-Efficacy, Diabetes Distress, and of A1c at 6 months. More home visits were significantly associated with higher self-efficacy. Self-efficacy at 6 months was a predictor of self-management behavior at 6 months. Although gender was significantly associated with attending Journey to Health and self-management classes in group format, neither of these variables predicted any of the outcomes. With an *n* = 123, results showed 90% power for linear regression.

### 3.4. Model Fit Results

The model produced an AGFI of 0.97 meaning that our model explained 97% of the generalized covariance among the predictors and outcomes. The model also produced a standardized root mean square residual (SRMR) of 0.05 which indicated that the model had a good predictive fit. Additionally, the model’s root mean square error of approximation (RMSEA) was 0.06 with a 90% confidence interval of (0.04, 0.08). This indicates that the model has an appropriate amount of parsimony. Lastly, the model’s CFI score was 0.96 indicating that the intervention model was a 96% improvement over the null model. The model’s LRT results indicated that the effects of removing demographics and treatment group and doctor visits were small.

### 3.5. Sample Demographic and Participation Characteristics by Gender

Table 1 summarizes the demographics, participation, and baseline measures of the sample. The ages of the women and men in the study were similar; participants were, on average, 49 years of age. About 61% of the sample was female. Men were more likely to be high school graduates, 40% of men compared to 24% of women, *p* = 0.013. On average, participants attended 7.6 out of 11 intervention classes, with no difference by gender. More men (62%) attended all healthy lifestyles classes in group format compared to women (43%), *p* = 0.023. Similarly, 73% of men attended all self-management classes in group format compared to 56% of all women, *p* = 0.032. Both men and women received an average of one CHW home visit and 84% had at least one CHW-accompanied doctor visit.

#### Baseline Measures and Medication Use

At baseline, 71% took oral diabetes medication and 24% used insulin. Approximately one third intensified their diabetes medication regimen from baseline to 6 months. The mean baseline A1c was 7.8, knowledge of diabetes management 2.8, self-efficacy 7.1, and self-management behavior 3.4. Only diabetes distress differed significantly between women (2.2) and men (1.9), *p* = 0.019.

### 3.6. Linear Regression Results

Linear regression was used to test for interactions between gender and intervention participation. Because the gender x participation interactions were not significant and the likelihood ratio tests indicated that the models fit better without the interactions, the gender interactions were removed and we report the models in Table 2a–e with only main effects.

Intervention class attendance was a significant predictor of Self-Efficacy, Diabetes Distress, and of A1c at 6 months. At 6 months, self-efficacy increased by an average of 0.06 (0.004, 0.12), *p* < *0*.05, diabetes distress dropped by −0.04 (−0.08, −0.01), *p* < *0*.01, and A1c dropped by −0.10 (−0.19, −0.02), *p* < *0*.05, for each intervention class attended. So, if a client attended 10 intervention classes, the average changes would a 0.6 increase in self-efficacy, −0.4 reduction in diabetes distress, and a −1.0 drop in A1c.

More home visits were significantly associated with higher self-efficacy. Self-efficacy at 6 months was a predictor of self-management behavior at 6 months. Although gender was significantly associated with attending Journey to Health and self-management classes in group format, neither of these variables predicted any of the outcomes.

With an *n* = 123, according to SAS Proc Power, the power for a linear regression model is over 90%. For the linear regression, power was calculated to detect a difference of 5% in the R2 from the linear regressions. The r-squares for each model ranged from 0.34 to 0.55, indicating that a significant proportion of variance was explained by the models.

## 4. Discussion

The current study modeled whether gender impacted the process of change among Latino/a’s with type 2 diabetes in a CHW-led intervention. Analysis showed no gender difference in the effect of the intervention on study outcomes. However, while gender was also not significant in linear regression results, we found that intervention class attendance was a significant predictor of self-efficacy, diabetes distress, and of A1c at 6 months. In addition, more home visits were significantly associated with higher self-efficacy.

While in bivariate analyses, men were significantly more likely to attend all healthy lifestyle and self-management classes in group format, our path analysis found no gender difference in the impact of group class on any of the outcomes. We hypothesize that offering classes during non-traditional work hours, supplementing transportation to and from the intervention site and holding classes in community-based locations may have increased class attendance [7,8,9,10]. Further, gender was not significant in any linear regression models and the models fit better without interactions between gender and participation. This aligns with a prior study of gender differences in a CHW-led diabetes lifestyle intervention; while not a path analysis, Hawkins et al. found that among Latino participants, men were less likely than women to complete the study, attend group classes, and complete CHW home visits [13]. Despite this fact, in the present study no gender differences were found in treatment outcomes such as glycemic control and diabetes self-management behaviors. These findings may mean that, while participation of men may be lower in CHW-led interventions, attendance to fewer intervention group classes still have a powerful impact on health outcomes. It is important to note, however, although gender was significantly associated with attending intervention classes in group format, class attendance did not predict any of the primary and secondary study outcomes (i.e., A1c). Future studies are needed that further investigate the mechanisms of change in CHW diabetes interventions, with a specific focus on group class attendance. While our study did not reveal gender differences in intervention outcomes (i.e., glycemic control and diabetes self-management behaviors), the intervention itself was significantly associated with improvements in diabetes self-efficacy, diabetes distress, knowledge (understanding of diabetes management), and A1c. These findings are consistent with prior work in Latino/a populations which show that use of culturally tailored diabetes education and lay helpers can significantly lower levels of diabetes distress which increasing knowledge of diabetes self-management behaviors [30,31,32,33].

Our path analysis found that higher self-efficacy scores were associated with better self-management. Specifically, self-efficacy was a predictor of self-management behavior at 6 months. This finding aligns with previous work which highlights a positive relationship between self-efficacy and diabetes self-management behaviors [33]. Qin and colleagues conducted a systematic review of existing literature (1990–2018) on the relationship between self-efficacy and diabetes self-management in adults in the United States and to determine whether the relationship applies across race and ethnicity [33]. Interestingly, they found that higher self-efficacy in Latinos predicted better self-management behaviors, but not for black and non-Hispanic white participants [30].

While our study did not disaggregate for Latino nationality/country of origin, previous work with Latinos has also shown the positive impact of participation in a CHW diabetes program on self-efficacy [3,34]. In our analysis, intervention class attendance was not only a significant predictor of self-efficacy, but also diabetes distress, and of A1c at 6 months post-intervention. Additionally, more home visits were associated with higher self-efficacy. It is important to note that REACH not only utilized group health education classes, but also individual follow up with CHWs both at home and during clinic visits. In home visits, CHWs assisted participants in setting patient specific goals and supporting their progress. During home visits, CHWs helped participants improve their patient–provider communication skills and facilitated necessary referrals to other service systems. While CHWs did not directly facilitate doctor visits, attendance to at least one visit provided CHWs for context regarding doctor-patient interactions. We hypothesize that this may have contributed to the effectiveness of the group format, however, more research is needed to further explain this relationship.

As stated, while REACH classes were offered during accessible hours (evening and weekend) in community-based locations and transportation was provided [7,8,9,10], additional barriers to attendance may be faced by Latino men. Considering the significant impact of programs such as REACH, which utilize CHWs to teach diabetes-related content, it is critical to find ways to increase class attendance and availability for both Latino/a men and women. This reveals a need for more interventions that target efforts to improve self-efficacy while also delivering culturally appropriate services. Interventions such as these have the potential to promote self-management behaviors and improve health outcomes for both Latino men and women on a larger scale.

Although limitations of this study exist (including the use of self-reported data and the lack of biomarker data, and the potential lack of generalizability of findings, among others), unlike other studies of the CHW model in Latino/a populations, we were able to show whether specific components of the intervention functioned differently based on gender with a focus on potential pathways of change. Additionally, our study is the first to use of path analysis in a CHW intervention targeting Latino/a with type 2 diabetes to simultaneously test the route through which change occurs in the intervention by gender.

## 5. Conclusions

This study provides empirical support and expands the literature by revealing the pathways of change in CHW-led and culturally tailored type 2 diabetes intervention that can have a significant effect on participant behaviors and health status outcomes. Although gender was the initial impetus for our study, the CHW model worked best for both groups. Among men and women, our data show the multiple mechanisms through which overall participation in the intervention is associated with positive physiological and psychosocial outcomes. The data show that at 6 months, participating in the intervention was associated with an increased sense of self-efficacy, a decreased level of diabetes distress, and an increased level of knowledge regarding type 2 diabetes self-management. In turn, higher levels of self-efficacy were linked to improved type 2 diabetes self-management behavior [20]. Finally, elevated levels of self-management behaviors were associated with lower A1c levels.

A starting premise of the CHW perspective is that it is necessary to move beyond the usual care medical model that was developed especially for acute medical conditions. In the traditional medical framework, health care professionals have the key authority in terms of diagnosing and planning medical protocols [35]. Experience demonstrates that this view is less effective for managing chronic conditions such as type 2 diabetes [35]. The CHW model directs attention to the idea that patients should be participants; patients understand their priorities and lifestyles and this knowledge is key for developing effective diabetes self-management plans. Bringing together a team of health care professions, including CHWs, along with patients, proves to be an effective approach. Additionally, the framework is particularly effective if the practices are culturally tailored to specific race and ethnic groups [8]. CHWs provide guidance regarding diabetes self-management, assist in setting goals, probe regarding what information patients need regarding diabetes, and assist in improving communication with health care providers. Collectively, these assumptions influenced the design of our study.

## Figures and Tables

**Table 1:** Baseline Values, Demographics, and Participation by Gender, Mean (sd) or *n* (%).

Characteristic	Women (*n* = 135)	Men (*n* = 87)	Total (*n* = 222)	*p*-Value for Women & Men
Demographic Characteristics				
Age, years	48.2 (10.3)	49.9 (11.0)	48.9 (10.6)	0.242 ^i^
High school graduate	33 (24.4%)	35 (40.2%)	68 (30.6%)	0.013 ^j^
Intervention Group	86 (63.7%)	63 (72.4%)	149 (67.1%)	0.178 ^j^
Diabetes Medication Characteristics				
Diabetes Medications				0.190 ^k^
No medications	8 (5.9%)	2 (2.3%)	10 (4.5%)	
Only oral diabetes medications	97 (71.9%)	61 (70.1%)	158 (71.2%)	
Insulin, with or without oral medication	30 (22.2%)	24 (27.6%)	54 (24.3%)	
				
Diabetes Med Intensification ^a^	40 (34.8%)	21 (30.4%)	61 (33.2%)	0.544 ^j^
				
Outcome Values at Baseline				
HbA1 ^c^	7.64 (1.78)	8.13 (1.99)	7.83 (1.88)	0.058 ^i^
Knowledge ^b^	2.76 (0.79)	2.84 (0.88)	2.79 (0.83)	0.493 ^i^
Diabetes Distress^c^	2.20 (1.02)	1.87 (0.94)	2.07 (1.00)	0.019 ^i^
Self-Efficacy ^d^	6.94 (1.72)	7.28 (1.56)	7.07 (1.67)	0.139 ^i^
Self-management behavior ^e^	3.43 (1.21)	3.42 (1.21)	3.42 (1.21)	0.944 ^i^
				
Intervention Participation Measures (*n* = 149)				
Characteristic	Women (*n* = 86)	Men (*n* = 63)	Total (*n* = 149)	*p*-Value for Women & Men
Class Attendance	8.0 (3.6)	7.1 (3.9)	7.6 (3.7)	0.113 ^l^
All Journey-to-Health ^f^ Classes in Group Format	37 (43.0%)	39 (61.9%)	76 (51.0%)	0.023 ^j^
All Self-Management ^g^ Classes in Group Format	48 (55.8%)	46 (73.0%)	94 (63.1%)	0.032 ^j^
Number of CHW ^h^ Home Visits	1.2 (1.3)	0.8 (1.0)	1.1 (1.2)	0.077 ^l^
At Least One CHW-Accompanied Doctor Visit	74 (86.0%)	51 (81.0%)	125 (83.9%)	0.403 ^j^

^a^ Diabetes Med Intensification = Higher dose or frequency of same diabetes medications or addition of new medications from baseline to 6 months calculated for those with 6-month data. ^b^ Knowledge of Diabetes management from Diabetes Care Profile Scale. ^c^ Diabetes Distress Scale. ^d^ Stanford Self-Efficacy for Diabetes Scale. ^e^ Summary of Diabetes Self-care Activities (On how many of the past 7 days did you …?) ^f^ Journey-to-Health = Part 1 of Diabetes Education Curriculum. ^g^ Self-Management = Part 2 of Diabetes Education Curriculum. ^h^ CHW = Community Health Worker. ^i^ T-test. ^j^ Pearson Chi-square Test. ^k^ Cochran–Armitage Trend Test. ^l^ Wilcoxon Test.

**Table 2:** a–e: Gender and Linear Regression on Cohort 3 Outcomes. a: 6-Month Self-Efficacy (Stanford Scale). R^2^ = 0.423. b: 6-Month Understanding of Diabetes Management (Diabetes Care Profile). R^2^ = 0.428. c: 6-Month Diabetes Distress. R^2^ = 0.338. d: 6-Month Self-Management Behavior (Summary of Diabetes Self-Care Activities Scale). R^2^ = 0.458. e: 6 Month HbA1c. R^2^ = 0.545.

Predictor	Coefficient (95% CI)	*p*-Value
Baseline Self-Efficacy (Stanford Scale)	0.61 (0.49, 0.74)	<0.001
Male gender referenced to female	0.24 (−0.18, 0.67)	0.262
Age (years)	0.00 (−0.014, 0.02)	0.615
High School Graduate	−0.01 (−0.44, 0.43)	0.972
Class attendance (0–11)	0.06 (0.00, 0.12)	0.037
All JTH classes in group format	0.14 (−0.37, 0.65)	0.597
All self-management classes in group format	0.44 (−0.10, 0.97)	0.112
CHW Home Visits	0.24 (0.01, 0.47)	0.042
Predictor	Coefficient (95% CI)	*p*-Value
Baseline Understanding of Diabetes Management	0.50 (0.36, 0.64)	<0.001
Male gender referenced to female	0.06 (−0.18, 0.30)	0.602
Age (years)	0.01 (0.001, 0.02)	0.037
High School Graduate	0.45 (0.19, 0.71)	0.001
Class attendance (0–11)	0.03 (−0.01, 0.08)	0.181
All JTH classes in group format	−0.07 (−0.32, 0.18)	0.560
All self-management classes in group format	0.05 (−0.20, 0.30)	0.687
CHW Home Visits	0.05 (−0.06, 0.16)	0.365
Predictor	Coefficient (95% CI)	*p*-Value
Baseline Distress	0.47 (0.35, 0.58)	<0.001
Male gender referenced to female	−0.08 (−0.31, 0.15)	0.502
Age (years)	0.00 (−0.01, 0.01)	0.682
High School Graduate	0.03 (−0.20, 0.27)	0.793
Class attendance (0–11)	−0.04 (−0.08, −0.01)	0.007
All JTH classes in group format	−0.27 (−0.55, 0.01)	0.059
All self-management classes in group format	−0.12 (−0.41, 0.16)	0.399
CHW Home Visits	−0.08 (−0.21, 0.04)	0.188
Predictor	Coefficient (95% CI)	*p*-Value
Baseline Self-Management Behavior	0.41 (0.27, 0.55)	<0.001
Male gender referenced to female	0.18 (−0.19, 0.56)	0.335
Age (years)	0.00 (−0.02, 0.02)	0.997
High School Graduate	0.37 (−0.05, 0.79)	0.083
Month 6 Baseline Knowledge of Diabetes Management	−0.26 (−0.52, 0.00)	0.053
Month 6 Diabetes Distress	−0.09 (−0.39, 0.20)	0.536
Month 6 Self-Efficacy	0.20 (0.04, 0.35)	0.017
Class attendance (0–11)	0.04 (−0.04, 0.11)	0.314
All JTH classes in group format	−0.18 (−0.57, 0.21)	0.366
All self-management classes in group format	−0.38 (−0.77, 0.00)	0.054
CHW Home Visits	0.14 (−0.03, 0.31)	0.113
Predictor	Coefficient (95% CI)	*p*-Value
Baseline HbA1c	0.59 (0.47, 0.71)	<0.001
Male gender referenced to female	−0.10 (−0.53, 0.33)	0.646
Age (years)	−0.01 (−0.03, 0.00)	0.141
High school graduate	−0.36 (−0.82, 0.09)	0.120
6-Month Self-Management Behavior	0.01 (−0.17, 0.19)	0.897
Medication Intensification	0.00 (−0.42, 0.42)	0.993
Class attendance (0–11)	−0.10 (−0.19, −0.02)	0.023
All JTH classes in group format	0.03 (−0.42, 0.48)	0.899
All self-management classes in group format	−0.19 (−0.64, 0.26)	0.402
CHW Home Visits	−0.06 (−0.26, 0.13)	0.539

## Data Availability

Data sharing not applicable. No new data were created or analyzed in this study. Data sharing is not applicable to this article.

## References

[1] Centers for Disease Control and Prevention National Diabetes Statistics Report 2020 Website. https://www.cdc.gov/diabetes/data/statistics-report/index.html.

[2] Greg E.W., Zhuo X., Cheng Y.J., Albright A.L., Narayan K.V., Thompson T.J. (2014). Trends in lifetime risk and years of life lost due to diabetes in the USA, 1985–2011: A modelling study. Lancet Diabetes Endocrinol..

[3] Palmas W., Findley S.E., Mejia M., Batista M., Teresi J., Kong J., Silver S., Fleck E.M., Luchsinger J.A., Carrasquillo O. (2014). Results of the northern Manhattan diabetes community outreach project: A randomized trial studying a community health worker intervention to improve diabetes care in Hispanic adults. Diabetes Care.

[4] Kim K., Choi J.S., Choi E., Nieman C.L., Joo J.H., Lin F.R., Gitlin L.N., Han H.R. (2016). Effects of community-based health worker interventions to improve chronic disease management and care among vulnerable populations: A systematic review. Am. J. Public Health.

[5] Shah M., Kaselitz E., Heisler M. (2013). The role of community health workers in diabetes: Update on current literature. Curr. Diabetes Rep..

[6] Kieffer E.C., Willis S.K., Odoms-Young A.M., Guzman J.R., Allen A.J., Feathers J.T., Loveluck J. (2004). Reducing disparities in diabetes among African-American and Latino residents of Detroit: The essential role of community planning focus groups. Ethn. Dis..

[7] Feathers J.T., Kieffer E.C., Palmisano G., Anderson M., Janz N., Spencer M.S., Guzman R., James S.A. (2007). The development, implementation, and process evaluation of the REACH Detroit partnership’s diabetes lifestyle intervention. Diabetes Educ..

[8] Spencer M.S., Kieffer E.C., Sinco B., Piatt G., Palmisano G., Hawkins J., Lebron A., Espitia N., Tang T., Funnell M. (2018). Outcomes at 18 months from a community health worker and peer leader diabetes self-management program for Latino adults. Diabetes Care.

[9] Two Feathers J., Kieffer E.C., Palmisano G., Anderson M., Sinco B., Janz N., Heisler M., Spencer M., Guzman R., Thompson J. (2005). Racial and ethnic approaches to community health (REACH) Detroit partnership: Improving diabetes-related outcomes among African American and Latino adults. Am. J. Public Health.

[10] Spencer M.S., Rosland A.-M., Kieffer E.C., Sinco B.R., Valerio M., Palmisano G., Anderson M., Guzman J.R., Heisler M. (2011). Effectiveness of a community health worker intervention among African American and Latino adults with type 2 diabetes: A randomized controlled trial. Am. J. Public Health.

[11] American Public Health Association. Community Health Workers. American Public Health Association Web Site. https://www.apha.org/apha-communities/member-sections/community-health-workers.

[12] Norris S.L., Chowdhury F.M., Van Le K., Horsley T., Brownstein J.N., Zhang X., Jack L., Satterfield D.W. (2006). Effectiveness of community health workers in the care of persons with diabetes. Diabetic Med..

[13] Hawkins J., Kieffer E.C., Sinco B., Spencer M., Anderson M., Ann-Marie Rosland A.M. (2013). Does gender influence participation? Predictors of participation in a community health worker diabetes management intervention with African American and Latino adults. Diabetes Educ..

[14] Rhodes S.D., Daniel J., Alonzo J., Duck S., García M., Downs M., Hergenrather K.C., Alegría-Ortega J., Miller C., Boeving Allen A. (2013). A systematic community-based participatory approach to refining an evidence-based community-level intervention: The HOLA intervention for Latino men who have sex with men. Health Promot. Pract..

[15] Rhodes S.D., Leichliter J.S., Sun C.J., Bloom F.R. (2016). The HoMBReS and HoMBReS Por un Cambio interventions to reduce HIV disparities among immigrant Hispanic/Latino men. MMWR Suppl..

[16] Hawkins J., Watkins D.C., Kieffer E., Spencer M., Piatt G., Nicklett E.J., Lebron A., Espitia N., Palmisano G. (2017). An exploratory study of the impact of gender on health behavior among African American and Latino men with type 2 diabetes. Am. J. Men’s Health.

[17] Mansyur C.L., Rustveld L.O., Nash S.G., Jibaja-Weiss M.L. (2015). Social factors and barriers to self-care adherence in Hispanic men and women with diabetes. Patient Educ. Couns..

[18] Toobert D.J., Hampson S.E., Glasgow R.E. (2000). The summary of diabetes self-care activities measure: Results from 7 studies and a revised scale. Diabetes Care.

[19] Polonsky W.H., Fisher L., Earles J., Dudl R.J., Lees J., Mullan J., Jackson R.A. (2005). Assessing psychosocial distress in diabetes. Diabetes Care.

[20] Beckerle C.M., Lavin M.A. (2013). Association of self-efficacy and self-care with glycemic control in diabetes. Diabetes Spectr..

[21] Fitzgerald J.T., Anderson R.M., Gruppen L.D., Davis W.K., Aman L.C., Jacober S.J., Grunberger G. (1998). The reliability of the diabetes care profile for African Americans. Eval. Health Prof..

[22] Fitzgerald J.T., Davis W.K., Connell C.M., Hess G.E., Funnell M.M., Hiss R.G. (1996). Development and validation of the diabetes care profile. Eval. Health Prof..

[23] Fisher R.A. (1942). The Design of Experiments.

[24] Kalbfleish J.D., Prentice R.L. (2011). The Statistical Analysis of Failure Time Data.

[25] Diggle P. (2002). Analysis of Longitudinal Data.

[26] West B.T., Galecki A.T., Welch K.B. (2014). Linear Mixed Models.

[27] SAS Institute (2012). The MIXED Procedure.

[28] Van Buuren S. (2007). Multiple imputation of discrete and continuous data by fully conditional specification. Stat. Methods Med. Res..

[29] Rubin D.B. (2004). Multiple Imputation for Nonresponse in Surveys.

[30] Hu J., Wallace D.C., McCoy T.P., Amirehsani K.A. (2014). A family-based diabetes intervention for Hispanic adults and their family members. Diabetes Educ..

[31] Vincent D., McEwen M.M., Pasvogel A. (2008). The validity and reliability of a Spanish version of the summary of diabetes self-care activities questionnaire. Nurs. Res..

[32] Baig A.A., Benitez A., Locklin C.A., Gao Y., Lee S.M., Quinn M.T., Solomon M.C., Sánchez-Johnsen L., Burnet D.L., Chin M.H. (2015). Picture good health: A church-based self-management intervention among Latino adults with diabetes. J. Gen. Intern. Med..

[33] Martinez-Vega I.P., Doubova S.V., Aguirre-Hernandez R., Infante-Castañeda C. (2016). Adaptation and validation of the Distress Scale for Mexican patients with type 2 diabetes and hypertension: A cross-sectional survey. BMJ Open.

[34] Qin W., Blanchette J.E., Yoon M. (2020). Self-Efficacy and diabetes self-management in middle-aged and older adults in the United States: A systematic review. Diabetes Spectr..

[35] Funnell M.M., Anderson R.M. (2004). Empowerment and self-management of diabetes. Clin. Diabetes.

